# Next-Generation Oral Delivery Systems: Phytosomal Hinokitiol Tablets via REGEMAT 3D Bioprinter-Based 3D Printing for Enhanced Bioavailability

**DOI:** 10.1155/sci5/6678786

**Published:** 2025-06-30

**Authors:** Turky Omar Asar, Ghada A. Milibary, Alshaimaa M. Almehmady, Waleed Y. Rizg, Raed I. Felimban, Fuad H. Alnadwi, Abdelsattar M. Omar, Khalid M. El-Say, Tarek A. Ahmed

**Affiliations:** ^1^Department of Biology, College of Science and Arts at Alkamil, University of Jeddah, Jeddah, Saudi Arabia; ^2^Department of Pharmaceutics, Faculty of Pharmacy, King Abdulaziz University, Jeddah, Saudi Arabia; ^3^Department of Medical Laboratory Sciences, Faculty of Applied Medical Sciences, King Abdulaziz University, Jeddah, Saudi Arabia; ^4^Center of Innovation in Personalized Medicine (CIPM), 3D Bioprinting Unit, King Abdulaziz University, Jeddah, Saudi Arabia; ^5^Department of Nuclear Engineering, Faculty of Engineering, King Abdulaziz University, Jeddah, Saudi Arabia; ^6^Department of Pharmaceutical Chemistry, Faculty of Pharmacy, King Abdulaziz University, Jeddah, Saudi Arabia

**Keywords:** 3D printing, anticancer therapy, hinokitiol, molecular docking, pharmacokinetics, phytosomes

## Abstract

The administration of hinokitiol, a natural bioactive compound with promising therapeutic potential, particularly against breast cancer, faces notable challenges related to its poor solubility and low bioavailability, limiting its clinical applications. The present study aimed to advance a previously developed liquid hinokitiol-loaded phytosomal formulation by incorporating it into 3D-printed oral tablets using a REGEMAT 3D Bioprinter, containing either pure drug or drug-loaded phytosomes to enhance the pharmacokinetic performance and therapeutic efficacy of this compound. The tablets were formulated using hydroxypropyl methylcellulose-based paste and subjected to comprehensive quality control tests, including weight variation, thickness, drug content, friability, and in vitro dissolution. Scanning electron microscopy revealed that the phytosome-loaded tablets had a denser, waxy-like appearance with fewer voids, which contributed to improved drug release profiles. Molecular docking and molecular dynamics simulations revealed strong and stable interactions between hinokitiol and target proteins, providing insight into the potential anticancer activity of this compound through hydrogen bonding with DNA guanine 19 and hydrophobic interactions with residues such as Trp-1510, Leu-1513, and Met-1533. In vitro dissolution experiments showed faster and more complete drug release from phytosome-loaded tablets compared with those containing pure hinokitiol. Pharmacokinetic evaluation in male Wistar rats revealed the superior performance of phytosome-loaded tablets, with a higher maximum plasma concentration and greater area under the curve. These results highlight the potential of 3D-printed tablets with hinokitiol-loaded phytosomes as a novel drug delivery system that significantly improves the bioavailability, drug release, and therapeutic efficacy of hinokitiol. The integration of nanotechnology and 3D printing in the present study offers a promising platform for enhancing the clinical utilization of bioactive compounds with poor solubility, such as hinokitiol.

## 1. Introduction

Hinokitiol, a natural tropolone compound derived from plants of the *Cupressaceae* family, has garnered notable attention due to its broad spectrum of pharmacological activities, including potent antimicrobial, antioxidant, anti-inflammatory, and anticancer properties [1]. These attributes make it a promising candidate for therapeutic applications across various disease states [2]. However, the clinical utilization of hinokitiol is hindered by several inherent limitations, such as poor aqueous solubility (1.12 ± 0.02 mg/mL), instability, and low systemic bioavailability [3, 4]. These challenges have restricted the formulation options and therapeutic efficacy of hinokitiol, necessitating innovative drug delivery systems to fully exploit its potential. Nanotechnology-based approaches have shown great promise in addressing the solubility and permeability limitations of hydrophobic compounds [5, 6]. Among these, the development of phytosomal nanoparticles has proven particularly effective [7]. Phytosomes, which are lipid-based nanocarriers, incorporate poorly soluble drugs into their lipid bilayers, improving the solubility, stability, and bioavailability of these drugs. Furthermore, the phytosomal structure facilitates enhanced oral absorption through the gastrointestinal tract, thereby improving systemic drug delivery and therapeutic outcomes [7].

Personalized medicine represents a significant transformation in healthcare in which treatments are customized to align with the distinct genetic makeup, environmental influences, and lifestyle choices of each patient [8, 9]. This approach is particularly crucial in managing complex diseases such as cancer, where traditional one-size-fits-all treatments often prove inadequate. In this context, 3D printing has emerged as a transformative technology, enabling the creation of customized drug delivery systems and medical devices that align with the specific needs of each patient [10, 11]. In cancer treatment, 3D printing allows for the precise assembly of personalized drug-loaded tablets, implants, and scaffolds, which can be designed to release therapeutic agents at optimal rates and target specific tumor sites [12, 13]. This technology also facilitates the development of patient-specific surgical planning models and tools, enhancing the accuracy and efficacy of oncological interventions. Beyond cancer, 3D printing is revolutionizing the management of various diseases by enabling the production of tailored prosthetics, orthotics, and even organ models for transplantation [14]. As the field of personalized medicine continues to evolve, 3D printing stands at the forefront, offering innovative solutions that improve patient outcomes and advance the possibilities of individualized healthcare.

Integrating 3D printing and nanotechnology offers a promising strategy to address challenges associated with poor drug solubility and limited bioavailability. This combination allows for developing personalized drug delivery systems tailored to specific therapeutic needs. Such technologies are particularly advantageous in the treatment of metabolic disorders, where precise dosing, targeted delivery, and controlled release are essential for effective disease management [15]. Combining nanocarriers with 3D printing technology, highly customizable and precisely engineered drug delivery systems can be developed. 3D printing facilitates the assembly of complex dosage forms with tailored release profiles, geometries, and compositions, enabling patient-specific treatments. This synergy is particularly beneficial in managing metabolic disorders, where precise dosing, controlled drug release, and targeted delivery are critical for optimal therapeutic outcomes [16]. For instance, 3D-printed tablets incorporating nanocarriers can ensure the sustained release of bioactive molecules, reducing dosing frequency and improving patient compliance [17]. This innovative pairing enhances the pharmacokinetics of challenging drugs and represents a promising step toward personalized medicine and more effective treatment of complex metabolic conditions.

In our recently published study, an optimized hinokitiol-loaded phytosome formulation demonstrated promising anticancer efficacy against breast cancer cells [18]. Specifically, the optimized hinokitiol-loaded phytosome formulation was prepared using a thin-film hydration method, where hinokitiol was complexed with a drug-to-phospholipid molar ratio of 1:3.68, cholesterol content of 14.71% relative to total phospholipids, deoxycholic acid content of 7.51% relative to total phospholipids, and a hydration medium pH of 4.2. This optimized formulation yielded a particle size of 138.4 ± 7.7 nm, a zeta potential of −18.43 ± 0.88 mV, and an entrapment efficiency (EE) of 99.7 ± 6.11%. The present study aimed to advance this approach by developing 3D-printed tablets containing a hinokitiol-loaded phytosome formulation. These tablets were compared with 3D-printed tablets containing pure hinokitiol to evaluate their relative performance in drug delivery and therapeutic potential. Molecular docking analysis was performed to investigate the binding affinity and interactions of hinokitiol with target proteins, providing deeper insights into its potential anticancer mechanisms. Additionally, the prepared tablets' surface morphology and internal structure were examined using scanning electron microscopy (SEM) to assess their quality and design precision. Comprehensive quality control and pharmacokinetic experiments were conducted to evaluate the drug release profile, stability, and bioavailability of the developed formulations, aiming to establish their effectiveness as a novel platform for targeted anticancer therapy.

## 2. Materials and Methods

### 2.1. Materials

Hinokitiol was obtained from AK Scientific (Union City, CA, USA). Phospholipon 90 G (a high-purity soy-derived phosphatidylcholine ≥ 94% derived from soy lecithin) was generously supplied by Lipoid GmbH (Koln, Germany). Ethanol, cholesterol, sodium deoxycholate, sodium hydroxide, potassium hydrogen phthalate, potassium dihydrogen phosphate, sodium bicarbonate, lactose anhydrous, polyvinyl pyrrolidone (PVP) K90 (molecular weight, 360,000 Da), and Methocel A15 LV (containing 27.5%–31.5% methoxyl groups) were sourced from Sigma-Aldrich (St. Louis, USA). Microcrystalline cellulose (Avicel PH-101) was procured from Winlab Laboratory Chemicals (Leicestershire, UK). Hydroxypropyl methylcellulose (HPMC; 4000 cp) was acquired from Spectrum Chemical Manufacturing Corporation (Gardena, CA, USA). Croscarmellose sodium (Ac-di-sol) was obtained from Biosynth International, Inc. (San Diego, CA, USA).

### 2.2. Preparation and Characterization of Hinokitiol-Loaded Phytosome Formulations

An optimized hinokitiol-loaded phytosome formulation was prepared and characterized for particle size, polydispersity index (PDI), zeta potential, and EE, following the methodology outlined in our previous study [18]. The optimized formulation was prepared using a drug-to-phospholipid molar ratio of 1:3.68. Cholesterol and sodium deoxycholate were included at 14.71% and 7.51%, respectively, relative to the total phospholipid weight. Briefly, 100 mg of hinokitiol, along with calculated amounts of Phospholipon 90 G (the primary amphiphilic phospholipid component), cholesterol, and sodium deoxycholate, was dissolved in 20 mL of ethanol using water bath sonication in a round-bottom flask. The ethanol was then evaporated under reduced pressure at 45°C using a Buchi Rotavapor R-200 (Buchi Labortechnik AG) until a thin lipid film formed inside the flask. To ensure the complete removal of the organic solvent, the flask was left overnight at 25°C in a vacuum oven (Thermo Fisher Scientific Inc.). The lipid film was subsequently hydrated with 50 mL of the specified hydration medium (buffer pH of 4.2), and the mixture was stirred at 50°C for 30 min. Finally, the mixture was homogenized at high speed (20,000 rpm) for 10 min to ensure uniform dispersion.

Particle size and PDI were measured using dynamic light scattering at a fixed angle of 173° (noninvasive backscatter) at 25°C. Before analysis, the samples were diluted 1:10 in deionized water to avoid multiple scattering effects. Measurements were recorded in triplicate, and average values were reported. Zeta potential was determined via electrophoretic light scattering using laser Doppler microelectrophoresis. Samples were similarly diluted and loaded into capillary cells. Measurements were conducted using the software (Zetasizer version 7.12; Malvern Panalytical Ltd.) to calculate zeta potential values.

An indirect method was used to estimate the percentage of hinokitiol entrapped within the phytosomal nanoparticles. A known volume of the prepared optimized formulation (*n* = 3) was centrifuged at 15,000 rpm for 60 min in Amicon Falcon tubes (Sigma Laboratory Centrifuge, Osterode, Germany). The supernatant was separated and filtered through a 0.22-μm filter, and the free drug content in the supernatant was determined by spectrophotometric analysis using a UV-Visible spectrophotometer (UV-1800, Shimadzu, Japan) at a wavelength of 241 nm, which corresponds to the maximum absorbance of hinokitiol in ethanol. A calibration curve was constructed using standard solutions of hinokitiol in ethanol within a concentration range of 2–20 μg/mL (*R*^2^ = 0.9995). The free drug concentration in the supernatant was quantified by interpolating the absorbance values against this standard curve. Each sample was analyzed in triplicate, and results were expressed as mean ± standard deviation (SD). The following equation was used to calculate the EE: EE = [(total amount of drug used − estimated amount of free drug in supernatant)/total amount of drug used] × 100.

### 2.3. Development of 3D Bioprinted Tablets

#### 2.3.1. Preparation of Hinokitiol-Loaded Pastes

HPMC (4%) was employed as a gelling agent to prepare two distinct gel formulations: phytosome-based hinokitiol and non–phytosome-based hinokitiol gels (pure drug-loaded gel). For the non–phytosome-based hinokitiol gel, a known weight of hinokitiol (100 mg) was dispersed in 100 mL distilled water using a magnetic stirrer, and 4 g of HPMC was gradually added to the mixture during stirring. For phytosome-based hinokitiol gel formulation, the same amount of HPMC was added to a specified volume of the optimized hinokitiol-loaded phytosome dispersion (equivalent to 100 mg hinokitiol) using a magnetic stirrer. The resulting two formulations were then stored at 4°C overnight to allow complete swelling of the polymer and the formation of a viscous gel free from air bubbles.

To develop the paste formulations, each gel formulation (phytosome or nonphytosome) was used as the base to which a powder blend comprising 8% (w/w) Avicel, 4% (w/w) PVP K90, 5% (w/w) lactose, 2.5% (w/w) Methocel, and 2.5% (w/w) Ac-Di-Sol was mixed and added to the mortar containing the prepared the final gel matrix. This blend was mixed until a smooth and uniform paste was produced. In these formulations, lactose, Methocel, and PVP K90 served as adsorbents; Ac-Di-Sol acted as a disintegrant; and Avicel functioned as an insoluble component. The selection of the formulation ingredients was based on our previously published study on the development of 3D-printed tablets containing glimepiride and/or rosuvastatin [19].

#### 2.3.2. 3D Bioprinting of Hinokitiol-Loaded Tablets

The formulated pastes were transformed into 3D-printed tablets using a REGEMAT 3D V1 BioPrinter (REGEMAT 3D) with computer-aided design modeling software (REGEMAT 1.4.9 Designer; Regemat 3D). Freshly prepared pastes were loaded individually into a 5-cc syringe and extruded through a 0.58-mm printing nozzle at a flow rate of 1.5 mm/s and a printing speed of 2.5 mm/s. The printing nozzle was adjusted to move in vertical and horizontal directions on the build plate at room temperature, printing the tablet structure layer by layer. Each tablet, consisting of five layers and measuring 9 mm in diameter, required an average of 10 min to print. After printing, the tablets were dried for a full day in a vacuum dryer at 40°C to ensure complete drying and fusion of the layers. The dried tablets were then stored in a tightly sealed container until further examination. The hinokitiol concentration in this formulation can be adjusted based on specific requirements.

### 2.4. Quality Control Tests for the Prepared Bioprinted Tablets

The dried 3D-printed tablets from both formulations were evaluated for weight variation, thickness, diameter, friability, content uniformity, and in vitro drug dissolution. The internal and surface structures were also investigated using a SEM.

#### 2.4.1. Weight Variation

To measure the weight variation, 10 tablets were individually weighed using an AJ100 electric balance (Mettler Toledo, LLC). The weight of each tablet was recorded, and the results were reported as the mean weight (mg) ± SD.

#### 2.4.2. Tablet Thickness and Diameter

The thickness and diameter of the tablets (*n* = 10) were determined using a dial indicator thickness gauge (Mitutoyo Corporation). The instrument provided precise measurements of both dimensions, ensuring consistency and quality control in the tablet manufacturing. The recorded measurements provided detailed data on the physical characteristics of the tablets, which are reported as the mean (mm) ± SD.

#### 2.4.3. Friability

The friability of the prepared tablets (*n* = 10) was evaluated using a Friabilator type PTF1, Pharma-test (Erweka GmbH). This test measures the tendency of tablets to crumble or break under mechanical stress. The tablets were subjected to rotation in the Friabilator for 4 min at 25 rpm. After the test, the tablets were reweighed, and friability was calculated as the percentage of weight loss from the original weight of the tablets using the following equation: Friability (%) = [(initial weight − final weight)/initial weight] × 100.

#### 2.4.4. Drug Assay

Five tablets from each formulation were individually placed in a mortar and crushed to ensure a uniform concentration of hinokitiol in each tablet across both formulations. Then, 100 mL of ethanol was added to each sample, and the mixture was filtered using filter paper to remove any solid particles. The resulting filtrate was analyzed using a UV–Vis spectrophotometer at 241 nm. The data obtained are reported as the percentage of hinokitiol content, expressed as mean ± SD.

#### 2.4.5. In Vitro Drug Dissolution

The in vitro drug dissolution of the prepared 3D-printed tablets (*n* = 3) was investigated using a paddle-type USP dissolution test apparatus (Type II), specifically the DT 700 LH device of Erweka GmbH. The test was conducted with 500 mL of distilled water maintained at 37°C, and the paddle speed was set to 50 rpm. Samples of 3 mL were taken at intervals of 5, 10, 15, 30, 45, 60, 90, and 120 min, with an immediate replacement to maintain the sink conditions. The drug content in each sample was measured spectrophotometrically at 241 nm against a blank of distilled water. The dissolution data are reported as mean (min) ± SD, providing a detailed drug release profile over time.

### 2.5. SEM Investigation

A SEM was utilized to examine the morphological characteristics and structural integrity of the prepared 3D-printed tablets. SEM images of the surface and inner structures were obtained using a Philips XL30 SEM (Philips Healthcare). The tablet samples were carefully sectioned using a surgical scalpel, mounted onto aluminum stubs, and sputter-coated with gold to enhance the conductivity and image quality. Imaging was performed at an accelerating voltage of 10 kV, allowing for detailed visualization of the tablets' microstructures.

### 2.6. Molecular Docking

Molecular docking of hinokitiol was performed to assess its binding affinity and interaction with target proteins associated with cancer therapy, DNA (cytosine-5)-methyltransferase 1 (DNMT1). This computational approach provided valuable insights into the potential mechanisms of action of hinokitiol at the molecular level, enabling the identification of key protein targets and binding sites. By predicting the strength and specificity of these interactions, molecular docking helped rationalize the compound's therapeutic potential, guiding the design of the formulation and experimental workflow. This step was essential for validating the feasibility of proceeding with in vivo pharmacokinetic studies, ensuring that the molecular behavior of the drug aligned with its intended therapeutic objectives.

#### 2.6.1. Preparation of Protein

For the docking assessment, the Protein Data Bank obtained the crystal structure of DNMT1 (PDB-ID: 6X9K). Before docking, the protein structure was prepared using the Schrödinger suite's Protein Preparation Wizard tool (including Protein Preparation Wizard, Epik, Impact and Prime; Schrödinger Release 2023-3; Protein Preparation Wizard; Epik, Schrödinger, LLC Inc., New York, NY, 2023; Impact, Schrödinger, LLC, New York, NY; Prime, Schrödinger, LLC, New York, NY, 2023). The preparation process included adding missing hydrogen atoms, correcting metal ionization states, and removing water molecules beyond 5 Å. Furthermore, appropriate charges were assigned, and the protein underwent restrained minimization using the OPLS4 force field to optimize its structural stability.

#### 2.6.2. Ligand Preparation

In this step, hinokitiol was converted from 2D to 3D structures using the LigPrep (Schrödinger Release 2023-3; Schrödinger, Inc.). Water molecules beyond 3 Å from hetero groups (HET), such as ligands or cofactors, were removed, and hydrogen bonds were optimized using PROPKA at pH 7.0. The OPLS4 force field was applied for restrained minimization, while metal HET states and cofactors were adjusted to a pH range of 7.0 ± 2.0.

#### 2.6.3. Receptor Grid Generation and Molecular Docking

Receptor grid generation and ligand docking were conducted using Glide (Schrödinger Release 2023-3). The grid box was defined by selecting the co-crystallized inhibitor (2R)-2-{[6-(4-aminopiperidin-1-yl)-3,5-dicyano-4-ethylpyridin-2-yl]sulfanyl}2-phenylacetamide (PDB-ID: UXM) as a reference, with the binding site established using the Receptor Grid Generation tool in Glide. The grid generated by Glide was then utilized for docking the prepared Hinokitiol ligands. The docking protocol selected was XP (extra-precision), with the van der Waals radii scaling factor set to 1.0 and a potential charge cutoff of 0.25. All other parameters were left at their default settings.

The molecular mechanics-generalized born surface area approach was employed using Prime (Schrödinger Release 2023–3: Schrödinger, Inc.) to re-score the docked ligand poses. The following equation was utilized to calculate the binding free energy (ΔG_bind_):

(1) $$\begin{array}{c} \Delta G_{\text{bind}}=E_{\text{Complex}}\left(\text{minimized}\right)-E_{\text{ligand}}\left(\text{minimized}\right)-E_{\text{protein}}\left(\text{minimized}\right), \end{array}$$

where “Complex” represents the protein–ligand complex, “E_protein_” corresponds to the free protein, and “E_ligand_” refers to the free ligands.

#### 2.6.4. Induced Fit Docking (IFD)

The IFD protocol was utilized to accurately model the flexibility of both the receptor and ligand using the IFD function in Maestro (v9.1; Schrödinger, Inc.) [20]. The complex was used to generate the centroid of the residues by selecting the ligand from the protein. The ligands were then docked into the protein using Glide.

### 2.7. Molecular Dynamic (MD) Simulation

MD simulations were conducted using the Schrödinger package, specifically the Desmond software package (including Desmond MDs System and Maestro-Desmond Interoperability Tools, Schrödinger Release2023; Schrödinger Inc.) [21]. The selected protein–ligand complexes were placed in a simple point charge water box, extended 10 Å beyond the atoms of the complex. System neutralization was achieved by adding Cl and Na counter ions. The simulation was performed at a 300 K temperature, with 1.01325 bar pressure. The force field was set at OPLS4 over 100 ns/trajectory, with the number of atoms, pressure, and temperature maintained constant. Figures and plots were sketched with the Maestro-Desmond simulation interaction diagram tool.

### 2.8. In Vivo Bioavailability Studies

The pharmacokinetics of the prepared 3D-printed tablets with hinokitiol-loaded phytosomes and those with pure hinokitiol were compared in the present study. Using a single oral dose of 600 μg/kg body weight for each animal, the study employed a one-period parallel design with male Wistar rats, each weighing 200–250 g. The rats were obtained from the animal house at the Faculty of Pharmacy, King Abdulaziz University (Jeddah, Saudi Arabia) and were housed under controlled conditions in the animal research facility. All animals were acclimatized prior to the study and handled in accordance with ethical guidelines. A total of 10 male Wistar rats were used and randomly divided into two groups (*n* = 5 per group): Group I received the 3D-printed tablets containing hinokitiol-loaded phytosomes, and Group II received the 3D-printed tablets containing pure hinokitiol. Before treatment, the animals were acclimated for 1 week in a temperature-controlled environment. All necessary animal welfare measures were implemented throughout the study to minimize pain and distress. Animals were housed under standard conditions with controlled temperature, humidity, and a 12-h light/dark cycle and were provided with free access to food and water. Moreover, animal health and behavior were monitored at least twice daily, once in the morning and once in the evening, throughout the acclimatization period and during the entire experimental duration.

The tablets were crushed and suspended in 0.5% carboxymethyl cellulose to prepare an oral drug suspension, administered using a flexible gastric tube designed for small laboratory animals. Specifically, a stainless-steel gavage needle with a rounded tip and a 1.5 mm diameter was used to ensure safe and precise administration. Blood samples (0.25 mL) were collected via the lateral tail vein from both groups at 1, 2, 4, 6, 12, and 24 h. At the end of the experiment, all animals were humanely euthanized using carbon dioxide inhalation at a displacement rate of 30%–70% of the chamber volume per min, in accordance with standard ethical guidelines. The plasma samples were immediately separated and stored at −20°C after centrifugation for 10 min at 6000 rpm.

For the preparation and extraction of the samples, a 1-mL aliquot of the blank, calibration standard, or unknown rat plasma sample was placed in a test tube. To each sample, 1 mL of the extraction working solution was added. The extraction working solution was prepared by mixing 0.1 M borate buffer (adjusted to pH 9.5 using NaOH) with a saturated Dabsyl chloride (Dabsyl-Cl) solution in acetonitrile in a ratio of 1:6. The mixture was subjected to vortex mixing for 1 min and centrifugation for 10 min at 5000 rpm. The organic phase was then separated and evaporated to dryness under a constant stream of nitrogen at 50°C. The residue was reconstituted in 80 μL of the mobile phase, and an injection volume of 20 μL was used. The HPLC method used for the quantification of Hinokitiol was adapted from a previously established method with slight modifications [22].

To determine the concentration of hinokitiol in the plasma samples, calibration standards were prepared using hydroethanolic stock solutions of the drug. The concentrations of hinokitiol in the unknown plasma samples and the calibration standards were measured using a Waters Alliance 2695 high-performance liquid chromatography (HPLC) instrument equipped with a variable wavelength UV detector and autosampler utilizing the data analysis software Empower 3 (Waters Corporation). Separation was achieved using an RP Hi-Q-Sil C18 column (150 × 4.6 mm, 5 μm) from Phenomenex Inc. The mobile phase consisted of water: acetonitrile: glacial acetic acid (69: 29: 2 v/v), with a flow rate of 1 mL/min. The UV detector was set at a wavelength of 447 nm.

The pharmacokinetic parameters of the 3D-printed tablets loaded with hinokitiol phytosomes and those with pure hinokitiol were evaluated. The analysis was conducted using a noncompartmental extravascular pharmacokinetic model via an Excel add-in program (PKsolver). Key parameters such as peak plasma concentration (*C*_max_) and the time to reach this concentration (*T*_max_) were quantified. Additionally, the areas under the curve (AUC; AUC_0–t_ and AUC_0–∞_) were determined to assess the overall drug exposure. Other pharmacokinetic parameters calculated included the mean residence time (MRT), the area under the first moment of the curve (AUMC_0-inf_), the elimination rate constant (*K*), and the half-life (t_1/2_). Furthermore, the apparent total body clearance (Cl) and volume of distribution (Vd) were also evaluated to provide a comprehensive understanding of the drug's pharmacokinetics.

The animal study protocol was approved by the Ethical Committee of the Faculty of Pharmacy at King Abdulaziz University (approval date: 31-01-2024; Approval No: TEMPORAR-19). The study adhered to the regulations set by the Food and Drug Administration, the European Medicines Agency, the International Conference on Harmonization, and Good Clinical Practice. Additionally, the “Standards of Laboratory Animal Care” ([National Institutes of Health (NIH) publication #8523, revised in 1985]) and the Guiding Principles in the Care and Use of Animals (Department of Health, Education, and Welfare DHEW publication, NIH #8023) were strictly followed throughout the research.

### 2.9. Statistical Analysis

The data obtained from the pharmacokinetics were statistically analyzed using GraphPad Prism Software version 9.5.1 (Dotmatics). A Sidak's multiple comparisons test was applied to compare each hinokitiol concentration at each time point between groups in the in vivo study. This test was used to assess the significance of the difference between groups, with *p* < 0.05 considered to indicate a statistically significant difference.

## 3. Results and Discussion

The development of the optimized Hinokitiol-loaded phytosomal liquid formulation was guided by a careful selection of formulation parameters to achieve the desired characteristics. The optimal combination of factor levels was identified, resulting in a drug-to-phospholipid molar ratio of 1:3.68, a cholesterol content of 14.71% relative to total phospholipids, a deoxycholic acid content of 7.51% relative to total phospholipids, and a hydration medium pH of 4.2 as mentioned in our recently published work [18]. These conditions produced a formulation with a particle size of 138.4 ± 7.7 nm, a zeta potential of −18.43 ± 0.88 mV, and a drug EE of 99.7 ± 6.11%. The morphological analysis confirmed the uniformity and spherical morphology of the optimized phytosomal nanoparticles, with no signs of aggregation or irregularities [18]. The well-defined spherical structure and smooth surfaces observed under microscopy indicate the successful formation of stable vesicles. These characteristics are crucial for ensuring consistent drug delivery and bioavailability, making this optimized formulation a promising candidate for further investigation in cancer therapy.

The primary objective of the present study was to translate the optimized liquid phytosomal formulation into a solid oral dosage form via 3D printing and compare its performance with 3D-printed tablets containing pure hinokitiol.

### 3.1. Quality Control Tests of the 3D Bioprinted Tablets

The quality control tests for the prepared 3D-printed tablets provided valuable insights into their physical and chemical characteristics. The 3D-printed tablets containing pure hinokitiol demonstrated an average tablet weight of 103 ± 11 mg, a 2.228 ± 0.224 mm thickness, and a 155 ± 11 mcg drug content. These values indicate good uniformity in the weight, thickness, and drug content of the tablet. It must be mentioned that the amount of drug contained in each tablet was selected for initial pharmacokinetic and in vitro studies. However, the drug dose can be easily adjusted by modifying the number of printed layers during the 3D printing process, allowing precise customization of dosage strength as needed.

The hinokitiol-loaded phytosomal 3D-printed tablets exhibited an average weight of 102 ± 8 mg, a thickness of 2.213 ± 0.071 mm, and a drug content of 171 ± 12 mcg. These results reveal a slightly lower SD in both weight and thickness of the phytosomal 3D-printed tablets compared with the pure hinokitiol 3D-printed tablets, indicating improved consistency and uniformity. Furthermore, the drug content in the phytosomal tablets was higher and more consistent, as indicated by the lower SD, suggesting a more efficient incorporation and distribution of hinokitiol within the phytosomal matrix.

Both the pure hinokitiol and the hinokitiol-loaded phytosomal 3D-printed tablets demonstrated excellent uniformity in diameter, measuring consistently at 9 mm. This uniformity in diameter was consistent with the specifications set during the printing process, which ensured that the tablets maintain a consistent size. The reliable uniformity in diameter across all tablets validates the reproducibility and precision of the 3D printing method used, underscoring the effectiveness of this technique in producing standardized pharmaceutical formulations. As these tablets are developed using 3D printing rather than direct compression, slight variations in surface texture may occur due to the layer-by-layer fabrication process.

The friability of the dried 3D-printed tablets was found to be < 1%, indicating excellent mechanical strength. This low friability value demonstrates that the tablets can withstand mechanical stresses without significant loss of material. These results highlight the robustness of the 3D-printed tablets, making them suitable for practical use in pharmaceutical applications.

Overall, the results of the quality control tests highlight the superior uniformity and drug content consistency of the hinokitiol-loaded phytosomal 3D-printed tablets compared with the pure hinokitiol tablets as illustrated in Table 1. This suggests that the phytosomal formulation may offer more reliable and consistent dosing, which is essential for achieving the desired therapeutic effects. These findings also underscore the importance of thorough quality control testing in the development and optimization of 3D-printed drug delivery systems.

### 3.2. In Vitro Drug Dissolution

**Figure 1:**
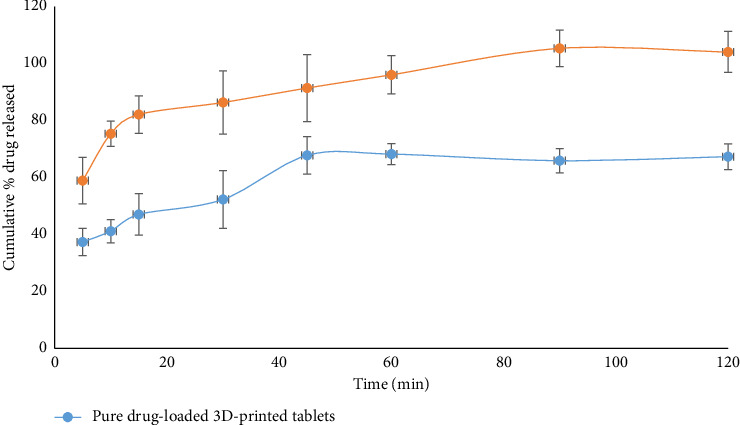
Dissolution profile of hinokitiol from the prepared 3D-printed tablet formulations (*n* = 3).

The in vitro drug dissolution profiles of both the pure hinokitiol and hinokitiol-loaded phytosomal 3D-printed tablets were evaluated and are represented in Figure 1. The dissolution profile of the hinokitiol-loaded phytosomal 3D-printed tablets was found to be superior compared with the pure hinokitiol tablets. This enhanced dissolution may be attributed to the small size of the phytosomes, which provide a larger surface area for drug release. This finding agrees with a previous study for the release of glimepiride and rosuvastatin 3D-printed tablets loaded with a self-nanoemulsifying drug delivery system [19].

The increased surface area facilitates a more efficient and faster release of hinokitiol from the phytosomal tablets into the dissolution medium. This suggests that the phytosomal formulation enhances the solubility and dissolution rate of hinokitiol, potentially leading to improved bioavailability. The superior dissolution profile of the phytosomal tablets indicates that this formulation may offer more consistent and effective therapeutic outcomes compared with the pure hinokitiol tablets. Beck et al. [23] developed 3D-printed tablets loaded with deflazacort polymeric nanocapsules and reported a faster release rate from the prepared tablet and attributed this behavior to enhanced drug solubility [23].

These findings underscore the advantage of using phytosomal formulations in improving the dissolution characteristics of poorly soluble drugs such as hinokitiol, thus optimizing their therapeutic efficacy. The enhanced dissolution profile is crucial for achieving rapid and consistent drug release, essential for effective treatment and patient compliance.

### 3.3. SEM

**Figure 2:**
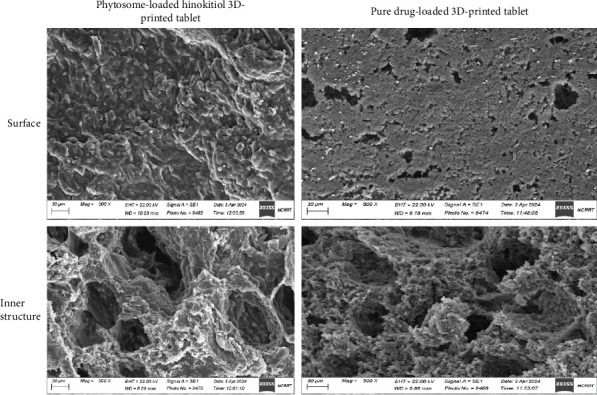
Scanning electron microscope images for the surface and inner structure of the prepared 3D-printed tablets.

The SEM images of the surface and inner structure of both the pure hinokitiol drug-loaded and hinokitiol-loaded phytosomal 3D-printed tablets are illustrated in Figure 2. The SEM images revealed distinct differences between the two tablet formulations. The surface of the pure hinokitiol tablets was relatively smooth but displayed a higher number of voids and pores, indicative of a less dense matrix. The inner structure of these tablets displayed a dried, interlocking flake-like structure that is attributed to tablet excipients [24], with some void spaces and pores. The interlocking flakes observed in the matrix are likely a result of the drying process, where excipients crystallized or aggregated in the absence of a stabilizing agent.

By contrast, the SEM images of the hinokitiol-loaded phytosomal tablets revealed a more cohesive and gel-like structure with significantly fewer voids and pores. The incorporation of the phytosomal formulation resulted in a denser matrix, which likely contributed to a slower rate of water loss during the drying process. Previous studies have demonstrated that phospholipids can self-assemble into a three-dimensional network, forming a gel-like structure that is expected to affect the prepared tablet characteristics [25]. This behavior is attributed to the cohesive matrix formed by the phospholipid components, which enhances moisture retention and imparts structural integrity even after the drying process. Such a matrix supports gradual swelling and erosion, leading to controlled drug release and reduced friability. Accordingly, the enhanced mechanical strength and superior dissolution profile observed in the phytosome-loaded tablets can be explained by the presence of this gel-like structure. This structural feature appears to aid in maintaining tablet integrity while controlling the drug release. Additionally, no visible drug crystals were observed on the surface of either tablet, a finding consistent with previous reports on diclofenac sodium 3D-printed tablets prepared via extrusion [26].

The SEM findings align with the mechanical and dissolution properties of the tablets. The denser, less porous inner structure of the phytosomal formulation provides improved mechanical strength and a more controlled dissolution profile, whereas the porous, flake-like nature of the pure hinokitiol tablets suggests a more rapid disintegration and dissolution process.

### 3.4. Molecular Docking

#### 3.4.1. Protein and Ligand Preparation

LigPrep was employed to transform the low-energy ionization and tautomeric states of 3D structures into their 2D representations for both hinokitiol and the co-crystallized ligands. Additionally, the DNMT1 (PDB-ID: 6X9K) was prepared via the Protein Preparation Wizard in which it underwent preprocessing, minimization of the geometry, and optimization of hydrogen bonds. Then, the generated structures were docked into each of the prepared PI3K-α and the docking scores were calculated.

#### 3.4.2. Validation of the Molecular Docking Results

**Figure 3:**
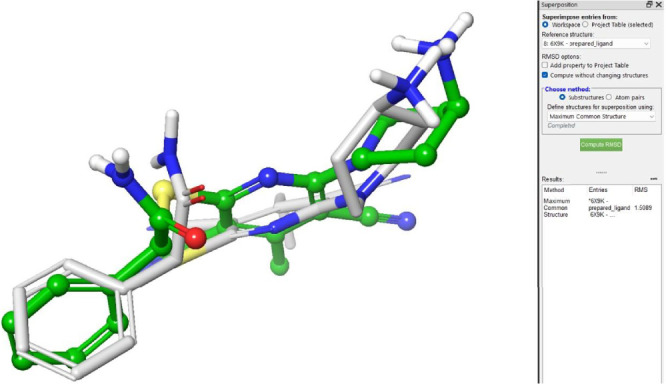
The 3D structure of the crystallized ligand redocked into the prepared DNA (cytosine-5)-methyltransferase 1 protein to validate the docking accuracy. The crystallographic pose is represented in green, whereas the predicted pose is presented in gray.

The choice of the docking site and the precision of the docking calculations were confirmed by re-docking the original ligand, which was used to create a 3D structure of the protein. The re-docking of the ligand resulted in a slight deviation, with a root mean square (RMS) value of 1.5 Å, demonstrating the reliability of the docking analysis (Figure 3).

#### 3.4.3. Molecular Docking Analysis

Following the setup of the grid box within the prepared DNMT1, which had been prepared using the Receptor Grid Generation tool in Maestro, the prepared 3D molecular structures were docked into the binding site of the prepared DNMT1, where the co-crystallized inhibitor was docked.  Table 2 shows the results of the docking of hinokitiol and the native inhibitor (PDB-ID: UXM) into DNMT1. These scores indicate the most favorable conformations of the bound ligands and their relative binding strengths. Hinokitiol showed good binding to DNMT1 compared with the native co-crystallized inhibitor. In addition to the docking score, other important scores were also calculated including the XP GScore, the Glide g-score, and the Glide Emodel. The XP docked complexes underwent assessment through the Xtra precision Glide score. This score fine-tuned the ligand binding energy by considering the force field parameters and penalties that played a significant role in the receptor-ligand binding. The IFD methodology by Schrödinger efficiently predicts active site geometries in protein–ligand complexes, addressing the challenge of protein conformation changes upon ligand binding. Utilizing Glide and Prime, IFD simulates the potential binding modes and subsequent conformational adjustments in the active site of the receptor. This process circumvents the need for time-intensive and often impractical crystallographic determinations, enabling rapid and cost-effective predictions [27].

Starting with Glide for initial docking, IFD generates various ligand poses by manipulating interaction parameters and selectively removing flexible side chains. Each pose undergoes refinement through Prime, which reorients adjacent side chains to enhance ligand accommodation. After minimization, the ligands are redocked into the optimized protein structures, and the complexes are evaluated using a combined scoring function of GlideScore and Prime energies. This approach ensures a comprehensive analysis of ligand binding potential, validated by real-world research applications and suitable for both novice and expert modelers [20].

**Figure 4:**
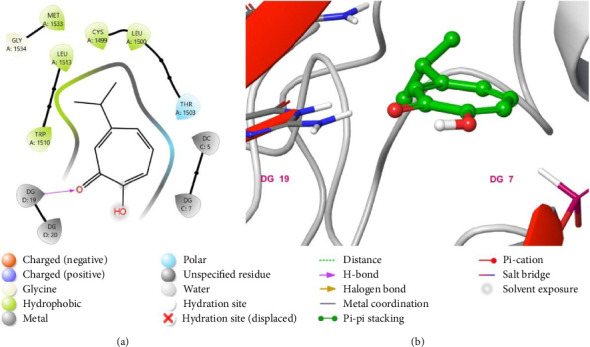
Putative binding mode of Hinokitiol at the binding site of DNMT1 receptor (PDB-ID: 6X9K). Hinokitiol is displayed as green sticks. Two-dimensional view (a); three-dimensional view of DNMT1 complexed with Hinokitiol (b).

The binding interactions between hinokitiol and DNMT1 were analyzed to identify the most significant interactions. The binding interactions between hinokitiol and the amino acid of the DNMT1(Figure 4) included a hydrogen bond between DNA guanin 19 and a carbonyl group of hinokitiol, and a group of hydrophobic interaction between the isopropyl group of hinokitiol and Trp 1510, Leu 1513, Cys 1499, Leu 1500, and Met 1533.

**Figure 5:**
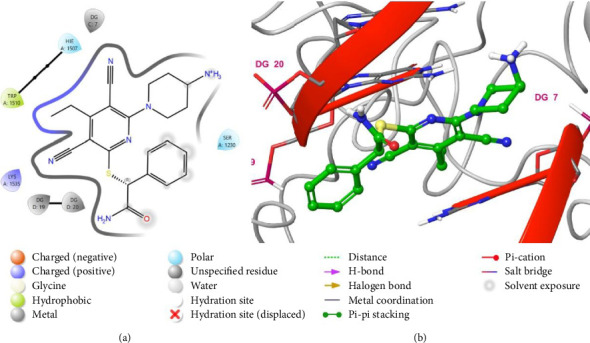
Putative binding mode of native agonist (PDB-ID: UXM) in the binding site of DNMT1 receptor (PDB-ID: 6X9K). UXM is displayed as green sticks. Two-dimensional view (a); 3D representation of DNMT1 complexed with UXM (b).

The binding interactions between the native inhibitor of DNMT1 (PDB-ID: UXM) and the receptor were also analyzed to identify the most important interactions (Figure 5). The interactions consisted of several hydrogen bonds. An interaction between DNA cytosine 5 (DC 5) and the NH_2_ group of the amide, an interaction between DC 22 and the amino group, and another interaction between Lys-1535 and the nitrile group. Additionally, two π-π stacking interactions were observed with the phenyl ring one between Tyr 1510 and the other with DC 5.

### 3.5. MD Simulations

MD simulations employ Newtonian physics to analyze atomic movements and explore the dynamic behavior of molecules in real-time [28]. MD simulation can capture various biomolecular processes, such as ligand binding, protein folding, and conformational changes, providing insights into their dynamic behavior [29]. The simulation was conducted under precisely controlled conditions. MD simulations were performed on the native agonist and the best binding agonists to validate the docking scores. The MD outcome of the native agonist was used as a control. The RMSD was employed to assess the conformational stability of the structures throughout the simulation by measuring the average atomic displacement relative to a reference structure [30].

**Figure 6:**
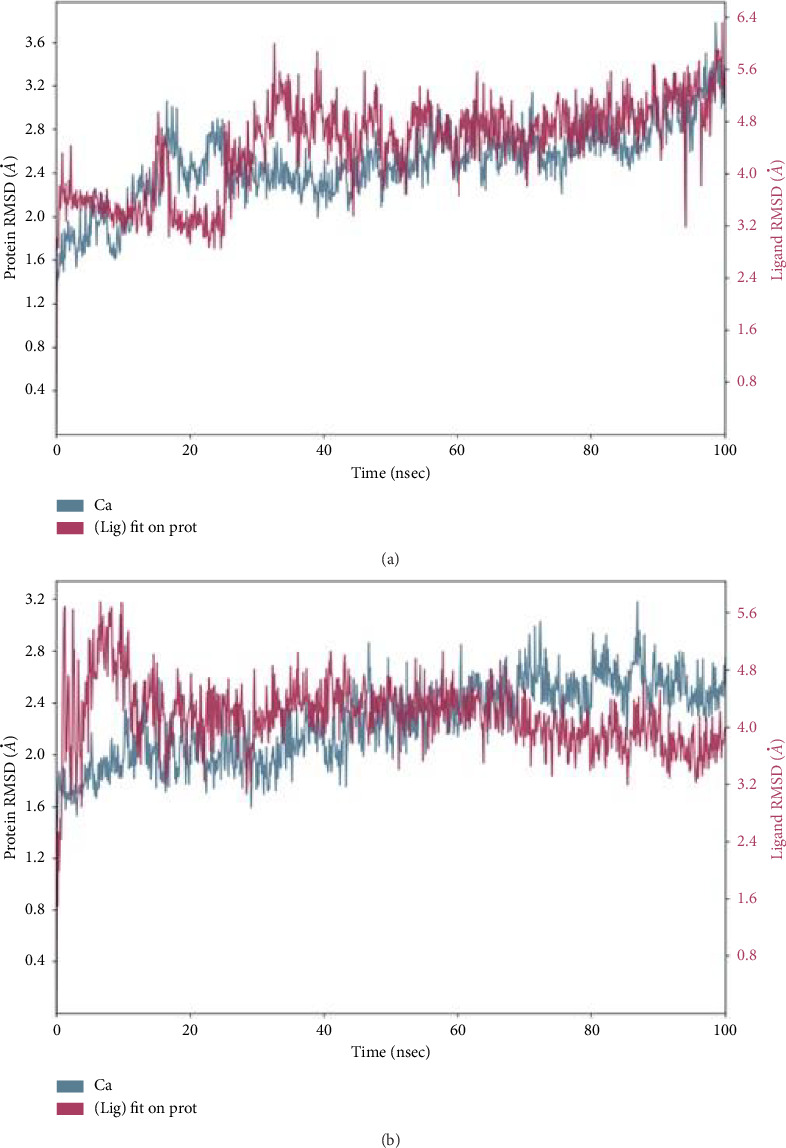
Root means square deviation (RMSD) of protein backbone atoms concerning the initial structure. DNMT1 (PDB-ID: 6X9K) bound to hinokitiol for 100 ns (a), DNMT1 (PDB-ID: 8EXL) bound to the co-crystallized ligand (PDB-ID: UXM) (b).

The RMSD plot in Figure 6(a) illustrates the RMSD evolution of the DNMT1 (left *y*-axis) aligned to the reference frame backbone, alongside the RMSD evolution of hinokitiol (right *y*-axis). The ligand RMSD indicates the stability of the ligand in relation to the protein. RMSD analysis aids in determining whether the simulation has reached equilibrium by evaluating fluctuations toward the end of the simulation, which should approximate a thermal average structure. As shown in the RMSD, although there were fluctuations during the simulation, they were within 1–3 Å, which is acceptable. The RMSD plot of the native inhibitor with the phosphoinositide 3-kinase (Figure 6(b)) also indicates the stability of the complex throughout the simulation with acceptable fluctuation within 1–3 Å.

**Figure 7:**
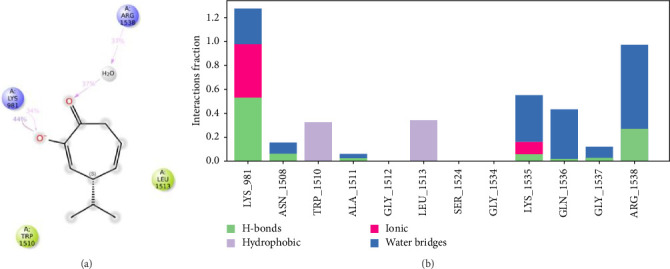
Schematic detailed representation of hinokitiol interactions with the DNMT1 (PDB-ID: 6X9K) residues, where violet represents the charged residues, and green represents the hydrophobic residues (a). Normalized stacked bar chart representing the interaction of the DNMT1 binding site residues with hinokitiol throughout the simulation: hydrophobic (violet), hydrogen bonds (green), ionic (red), and water bridges (blue). The stacked bar charts are normalized throughout the trajectory (b).

**Figure 8:**
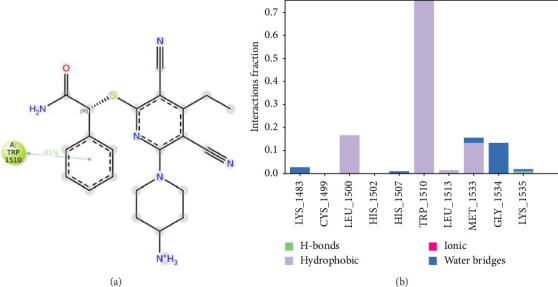
Schematic detailed representation of the co-crystallized ligand (PDB-ID: UXM) interactions with the DNMT1 (PDB-ID: 6X9K) residues, where violet represents the charged residues, and green represents the hydrophobic residues (a). Normalized stacked bar chart representing the interaction of the DNMT1 binding site residues with hinokitiol throughout the simulation: hydrophobic (violet), hydrogen bonds (green), ionic (red), and water bridges (blue). The stacked bar charts are normalized throughout the trajectory (b).

Throughout the simulation, one can track protein interactions with the ligand. These interactions can be classified by type and presented in summary, as illustrated in Figures 7 and 8. Protein–ligand interactions, also known as “contacts,” fall into four categories: hydrogen bonds, hydrophobic interactions, ionic interactions, and water bridges. The stacked bar charts are adjusted for normalization across the entire trajectory. The binding interactions between hinokitiol and the DNMT1 residues are presented in Figure 7. The most prominent interaction is the phenolic group with Lys 981, with > 125% of the trajectory time, and the second interaction between the ketone group and Arg 1538 with 98% of the trajectory time. The isopropyl group interacts through hydrophobic interactions with Trp-1510 and Leu-1513 with a trajectory simulation time of 30% and 35%, respectively.

The binding interactions of the native inhibitor (PDB-ID: UXM) with DNMT1 were also analyzed (Figure 8). These interactions were mainly hydrophobic interactions between the phenyl ring and Trp-1510 with 75% of the trajectory time.

Accordingly, the MD simulations provided crucial insights into the stability and interaction dynamics of hinokitiol and DNMT1, as well as the native inhibitor complex. The RMSD analysis demonstrated that both the hinokitiol–DNMT1 complex and the native inhibitor complex maintained stable conformations throughout the simulation, with acceptable fluctuations within 1–3 Å, indicating equilibrium and structural stability. Overall, the MD simulation results validated the computational docking outcomes and provided a deeper understanding of hinokitiol molecular interactions, paving the way for further in vivo pharmacokinetic and therapeutic evaluations.

### 3.6. Pharmacokinetic Study

**Figure 9:**
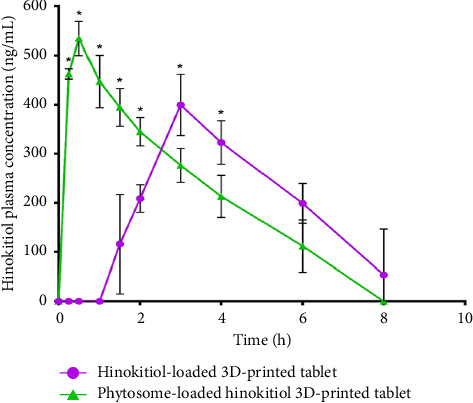
Plasma level–time curve for hinokitiol-loaded 3D-printed tablet and phytosome-loaded drug 3D-printed tablet after oral administration to male Wistar rats (*n* = 5). Note: ^∗^indicates a significant difference between groups at *p* < 0.05.

In the present study, each animal received a single 3D-printed tablet containing 155–171 μg of hinokitiol, corresponding to a dose of 600 μg/kg based on the average body weight of the animals. This dosing strategy was chosen to ensure consistency across groups while maintaining a clinically relevant exposure level for evaluating the bioavailability of the formulations. Administering a whole tablet per animal also mimics a practical oral dosing scenario, allowing for a more accurate assessment of the pharmacokinetic performance of the phytosomal and pure hinokitiol tablets. Male rats were chosen to minimize potential variability in pharmacokinetic parameters that could arise due to hormonal fluctuations in female animals, which are known to influence drug metabolism. This approach ensures consistency in data interpretation and aligns with standard pharmacokinetic study designs [31]. The pharmacokinetic parameters calculated for the pure hinokitiol 3D-printed tablets and the hinokitiol-loaded phytosomal 3D-printed tablets are presented in Table 3. The plasma concentration–time curves following oral administration of both tablet formulations are depicted in Figure 9. The hinokitiol-loaded phytosomal tablets demonstrated a higher Cmax compared with the pure hinokitiol tablets, indicating enhanced drug oral absorption. This improvement in oral absorption is likely due to the nano-sized phytosomal formulation of hinokitiol, which increases the surface area and facilitates improved absorption in the gastrointestinal tract, as supported by previous studies [32–34].

Notably, the Tmax differed significantly between the two formulations. The hinokitiol-loaded phytosomal 3D-printed tablets achieved a Tmax of 0.5 h, whereas the pure hinokitiol 3D-printed tablets reached Tmax at 3 h. This notable difference can be attributed to the enhanced solubility and oral absorption of the phytosomal nanodrug formulation, which facilitated faster drug release and uptake compared with the pure drug formulation. Baek et al. [35] investigated the dissolution and oral absorption of pranlukast suspensions and found that both the dissolution rate and oral absorption were significantly influenced by the particle size, which may account for the slightly higher Tmax of the phytosomal tablet.

The AUC, which reflects the overall body exposure to the drug, was higher for the phytosomal tablets than the pure hinokitiol tablets. This indicates that the phytosomal formulation improved the absorption rate and the extent of oral absorption. Conversely, AUMC, which is related to the MRT of the drug in the body, was higher for the pure hinokitiol 3D-printed tablets.

These results highlight the advantages of the hinokitiol-loaded phytosomal 3D-printed tablets regarding both the rate and extent of oral drug absorption. The superior pharmacokinetic profile of the phytosomal formulation suggests its potential for achieving more effective and consistent therapeutic outcomes compared with pure drug tablets. The present study focused on evaluating the pharmacokinetic advantages of the hinokitiol-loaded phytosomal tablets, demonstrating enhanced bioavailability and sustained drug release. Future studies will investigate the therapeutic efficacy and compare tumor reduction rates using a female breast cancer model to validate the clinical potential of the formulation further. In addition, comprehensive stability studies under ICH-recommended storage conditions and histopathological evaluations will be conducted to assess the long-term integrity and safety of the 3D-printed formulations. The absence of female animals in the present study represents a limitation, as sex-related differences could influence the pharmacological response.

## 4. Conclusion

The present study successfully developed and evaluated 3D-printed tablets containing hinokitiol in two forms: pure hinokitiol and phytosome-loaded nanoparticles. Comprehensive quality control tests confirmed the prepared tablets' uniformity, mechanical strength, and suitability for pharmaceutical use. SEM characterization revealed a denser, gel-like microstructure in the phytosomal tablets, improving drug release profiles by minimizing voids and enhancing control over dissolution. Molecular docking and MD simulations provided computational insights into the binding interactions of hinokitiol with the target protein, DNMT1, suggesting potential binding stability. While these in silico findings support the affinity of hinokitiol for DNMT1, further experimental validation is necessary to confirm its biological mechanism of action in breast cancer therapy. The pharmacokinetic experiments demonstrated that the hinokitiol-loaded phytosomal tablets significantly outperformed the pure hinokitiol tablets, showing enhanced bioavailability, higher Cmax, and greater AUC. These findings underscore the benefits of integrating nanotechnology with 3D printing to overcome the solubility and bioavailability challenges of bioactive compounds such as hinokitiol. This approach highlights the transformative potential of combining advanced formulation techniques with innovative manufacturing technologies to address critical challenges in drug delivery and enhance the clinical utility of poorly soluble compounds.

## Figures and Tables

**Table 1:** Quality control parameters of 3D-printed tablets containing pure hinokitiol and phytosome-loaded hinokitiol.

Parameter	Pure hinokitiol tablets	Phytosomal-loaded hinokitiol tablets
Average weight (mg)	103 ± 11	102 ± 8
Thickness (mm)	2.228 ± 0.224	2.213 ± 0.071
Diameter (mm)	9.00 ± 0.00	9.00 ± 0.00
Drug content (μg/tablet)	155 ± 11	171 ± 12
Friability (%)	0.83 ± 0.06	0.78 ± 0.05

**Table 2:** Docking results of in silico screening of hinokitiol against DNMT1 (PDB-ID: 6X9K).

Title	Docking score	XP GScore	Glide g-score	Glide Emodel	IFD score
6X9K–UXM prepared_ligand	−7.742	−7.747	−7.747	−75.338	−1925.74
Hinokitiol	−5.491	−5.935	−5.935	−24.767	−1921.10

**Table 3:** Pharmacokinetic parameters of hinokitiol after oral administration of 600 μg/kg in rats (*n* = 5, the data expressed as average ± SD).

Parameter	Unit	Hinokitiol phytosome-loaded 3D-printed tablet		Hinokitiol-loaded 3D-printed tablet	
		Average	STDEV	Average	STDEV
*K*	1/h	0.33	0.14	0.23	0.03
*t*_1/2_	h	2.42	1.09	3.00	0.34
*T*_max_	h	0.50	0.00	3.00	0.00
*C*_max_	ng/mL	534.67	34.70	399.67	62.56
AUC_0–8_	ng/mL ∗ h	1704.33	190.12	1531.92	416.12
AUC_0-inf_	ng/mL ∗ h	2449.89	577.52	2285.67	499.29
AUMC_0-inf_	ng/mL ∗ h^2^	8579.90	5340.26	14133.22	4107.52
MRT_0-inf_	h	3.76	1.30	6.12	0.46
VD	(mg/kg)/(ng/mL)	0.95	0.21	1.16	0.15
CL	(mg/kg)/(ng/mL)/h	0.24	0.07	0.27	0.06

*Note:* K, elimination rate constant; *t*_1/2_, elimination half-life; *T*_max_, the time point to reach the maximum plasma concentration; *C*_max_, the maximum plasma concentration over the time specified; AUC_0–8_, the area under curve from zero time to the last measurable concentration; AUC_0-inf_, the area under the plasma concentration–time curve from time zero to infinity; AUMC_0–inf_, the area under the first moment of the plasma concentration–time curve was from time zero to infinity; MRT_0–inf_, the mean residence time; VD, the apparent volume of distribution; CL, the total body clearance. AUC_0–t_ was calculated using the linear trapezoidal method. AUC_0–inf_ was estimated as the sum of the AUC_0–t_ plus the ratio of the last measurable plasma concentration to the elimination rate constant. MRT_0–inf_ was calculated from the ratio of AUMC to AUC. *t*_1/2_ was calculated as 0.693/K. CL was calculated by dividing the dose by AUC. VD was calculated by multiplying the total body clearance by MRT.

## Data Availability

The data generated in the present study are included in this article's figures and/or tables.
